# French youth trends in prevalence of overweight, obesity and underweight from 2008 to 2018

**DOI:** 10.1186/s13104-022-06091-3

**Published:** 2022-06-15

**Authors:** Jérémy Vanhelst, Jean-Benoît Baudelet, David Thivel, Hervé Ovigneur, Thibault Deschamps

**Affiliations:** 1 INFINITE-Institute for Translational Research in Inflammation (U-1286), Université de Lille, CHU Lille, Inserm, 59000 Lille, France; 2 Congenital & Pediatric Heart Unit, Institut Cœur Poumon, Univ. Lille, CHU Lille, 59000 Lille, France; 3 AME2P Laboratory, CRNH Auvergne, Univ. Clermont Auvergne, 63000 Clermont-Ferrand, France; 4 Diagnoform, IRFO, 59139 Wattignies, France

**Keywords:** Prevalence, Obesity, Health, Adolescence

## Abstract

**Objective:**

Because the prevalence overweight and obesity remains high during adolescence in Europe, tracking weight status in children and adolescents is needed. We aimed to estimate French trends in the prevalence of weight status in children and adolescent from 2008 to 2018.

**Results:**

The prevalence of overweight and obesity were higher in boys (P < 0.0001). Underweight was more prevalent in girls (P < 0.0001). In adolescents aged 16–17 years old, a stabilization was observed for overweight and obesity whereas the prevalence of underweight increased significantly in boys (P < 0.0001). For children and adolescents aged to 10–12 and 13–15 years old, the obesity and overweight decreased significantly while the underweight was increased for both sexes aged 10–12 years (P < 0.0001). Underweight increased in boys aged 13–15 years (P < 0.0001) while that a stagnation was observed in girls. These encouraging results show the permanent need to develop preventive strategies promoting an healthy active living in order to modify the lifestyle for adolescents with underweight, overweight or obesity.

## Introduction

Since many decades, obesity in children and adolescents is became a major childhood health problem in worldwide. In a recent systematic review and meta-analysis, authors showed the prevalence of overweight and obesity in youth were very high, but with a trend to stabilisation in most European countries [1]. Conversely, underweight becomes another health concern since last years, in many countries. Studies suggested that the prevalence of underweight tends to increase in developed countries [2–5]. Overweight, obesity and underweight is related to numerous health problems that tend to track from childhood into adulthood with considerable long-term health and economic burden.

Because the prevalence overweight and obesity remains high during adolescence, and in order to assess the effectiveness of interventions and public health recommendations developed by health policy markers, tracking weight status in children and adolescents is needed. Therefore, trends in children’s and adolescents' weight status have to be regularly documented in each country. Recently, authors published results regarding the prevalence of underweight, overweight and obesity in French children, underlying a stability of the prevalence of obesity, while the prevalence of overweight decreased significantly, remaining however high in French children [5]. Data on the prevalence rates in overweight and obesity in French adolescents are still lacking [6–8]. Although the prevalence of underweight shows a worrying upward trend in many developed countries, few data are also available in France [8, 9]. To our knowledge, data have been missing regarding the tracking of French adolescents’ weight status (underweight, overweight and obesity) since 2013 [7, 8].

The purpose of the present study was to analyse the secular trends in weight status (underweight, overweight and obesity) among French children and adolescents from 2008 to 2018.

## Main text

### Methods

Data from our study were provided from the French health program “Diagnoform^®^” (https://irfo.fr/) from 2008 to 2018. The main objective of this program was to assess the physical fitness levels in French children, adolescents and adults. For the children and adolescence population, measures were performed in the school environment (such as school playgrounds or sports club gymnasiums).

This study did not involve any intervention, and was conducted on a volunteer basis. Data were retrospectively collected by the study organizational structure (https://irfo.fr/). In this context, written informed consent was not required according to French human research regulations. However, the aims and objectives of the event were explained carefully to each adolescent and to their parents. All answers provided by parents were anonymous and confidential. This data collection was approved by the French National Commission of the Informatics Personal Data.

From this national database of Diagnoform program, a total of 90 250 children and adolescents aged 10–17 years were included in the present analysis.

### Measurements

#### Anthropometric measures

Physical measurements including weight, height and body mass index (BMI) were collected by the field workers from the organizer. Investigators asked to adolescents to complete a self-reported weight and height using a questionnaire. BMI was calculated as weight/height squared (kg/m^2^). Underweight, obesity and overweight prevalence were calculated according to International Obesity Task Force (IOTF) cut-offs [10]. These international cut-offs are defined by values of BMI at age 18: BMI 25 (overweight), 30 (obesity) and 18.5 (thinness).

Due to rapid change in anthropometric parameters during childhood and adolescence, age categories were created. Three categories were defined: (i) childhood 10–12 years; (ii) early adolescence (13–15 years) and (iii) late adolescence (16–17 years).

#### Statistical analysis

The data are presented as percentages for categorical variables and as means and standard deviations for continuous variables. The Shapiro–Wilk test was used in order to verify the normality of distribution.

Chi-squared test was used to compare prevalence means of underweight, overweight and obesity between boys and girls. Trends from 2007 to 2018 for underweight, overweight and obesity were assessed by the Cochran–Armitage trend test.

All statistical tests were performed at the two-tailed ∝ level of 0.05. Data were analysed using the statistical software packages IBM SPSS Statistics for Windows (Version 22.0; IBM SPSS, Armonk, NY, USA) R Project for Statistical Computing (v. 3.6.1) and Excel 2013 (Microsoft, Redmond, WA, USA).

## Results

The mean age was 13.9 ± 2.1 and 13.8 ± 2.8 years for boys and girls, respectively. Anthropometric measures and prevalence rates of each weight status (underweight, overweight and obesity) by sex and age group are presented in Table 1. Prevalence rates of overweight and obesity were significantly higher in boys compared than girls (P < 0.0001) (Table 1). For the underweight, the rate was also higher in girls than boys (P < 0.0001).

**Table 1:** Prevalence rates of underweight, overweight and obesity, and mean anthropometric characteristics with standard deviations in French boys and girls, age 10–17 years, during the period 2008–2018 (n = 90 250)

	Boys				Girls				P*
	10–12 years	13–15 years	16–17 years	Total	10–12 years	13–15 years	16–17 years	Total	
Underweight									
Prevalence (n/%)	2064/42.36	1860/38.17	949/19.47	4873/10.20	2543/42.04	2327/38.47	1179/19.49	6049/14.24	** < 0.0001**
Height (cm)	147.79 ± 8.45	165.34 ± 10.49	174.49 ± 8.00	159.69 ± 14.13	148.88 ± 8.52	162.05 ± 7.06	164.32 ± 6.48	156.95 ± 10.29	** < 0.0001**
Weight (kg)	31.61 ± 4.07	43.48 ± 6.86	51.58 ± 5.44	40.03 ± 9.55	32.32 ± 4.39	42.80 ± 4.87	46.27 ± 4.25	39.07 ± 7.44	** < 0.0001**
BMI (kg.m^2^)	14.42 ± 0.78	15.80 ± 0.94	16.90 ± 0.89	15.43 ± 1.28	14.52 ± 0.82	16.25 ± 0.95	17.11 ± 0.84	15.69 ± 1.36	** < 0.0001**
Overweight									
Prevalence (n/%)	2876/45.04	2336/36.58	1174/18.38	6386/13.37	2480/47.54	1567/33.15	681/14.41	4728/11.13	** < 0.0001**
Height (cm)	152.33 ± 8.74	169.64 ± 9.51	175.90 ± 7.65	163 ± 13.28	152.44 ± 8.09	161.79 ± 6.92	162.89 ± 6.60	157.05 ± 8.94	** < 0.0001**
Weight (kg)	53.05 ± 7.48	71.44 ± 9.59	80.85 ± 8.18	64.89 ± 14.03	53.59 ± 7.3	66.56 ± 7.06	70.02 ± 6.96	60.25 ± 10.08	** < 0.0001**
BMI (kg.m^2^)	22.75 ± 1.4	24.72 ± 1.44	25.62 ± 1.56	24.08 ± 1.92	22.96 ± 1.47	25.37 ± 1.50	26.34 ± 1.44	24.25 ± 2.03	** < 0.0001**
Obese									
Prevalence (n/%)	772/46.65	596/36.01	287/17.34	1655/3.46	673/55.85	348/28.88	184/15.27	1205/2.84	** < 0.0001**
Height (cm)	153.07 ± 9.33	169.17 ± 9.87	174.70 ± 8.56	162.62 ± 13.1	153.43 ± 8.68	161.66 ± 6.90	162.56 ± 7.77	157.21 ± 9.11	** < 0.0001**
Weight (kg)	66.78 ± 10.83	88.53 ± 13.47	99.48 ± 12.82	80.28 ± 17.94	67.84 ± 11.08	83.10 ± 10.85	87.07 ± 11.89	75.19 ± 13.92	** < 0.0001**
BMI (kg.m^2^)	28.37 ± 2.94	30.80 ± 2.76	32.53 ± 3.04	29.97 ± 3.31	28.71 ± 3.39	31.73 ± 3.14	32.94 ± 3.8	30.23 ± 3.81	0.05781
Normal weight									
Prevalence (n/%)	13,692/39.07	13,903/40.08	7270/20.85	34,865/72.97	11,980/39.29	12,821/42.05	5688/18.66	30,489/71.79	** < 0.0001**
Height (cm)	149.37 ± 8.60	169.24 ± 9.49	175.38 ± 6.92	162.72 ± 13.98	150.69 ± 8.62	162.38 ± 6.65	163.65 ± 6.40	158.02 ± 9.51	** < 0.0001**
Weight (kg)	39.65 ± 6.35	56.23 ± 8.76	63.94 ± 7.26	51.33 ± 12.4	40.81 ± 6.61	52.41 ± 6.28	55.52 ± 6.01	48.43 ± 8.91	** < 0.0001**
BMI (kg.m^2^)	17.68 ± 1.54	19.52 ± 1.69	20.75 ± 1.66	19.05 ± 2.02	17.87 ± 1.63	19.84 ± 1.68	20.71 ± 1.67	19.23 ± 2.01	** < 0.0001**

^*^The χ^2^ was performed to assess differences in prevalence rates by sex; Student’s t test was performed to assess differences in anthropometric data by sex. Significant P values are indicated in bold font

Percentages are row percentages for prevalence rates by age group; percentages are column percentages for total prevalence rates by sex

Table 2 shows trends in underweight, overweight and obesity according to sex and age between 2008 and 2018. In overall, significant changes in overweight, obese and underweight were found for boys and girls, respectively (P < 0.0001) (Table 2). The prevalence of overweight and obese was lower in 2018 than in 2008 in both sexes (P < 0.0001) (Table 2). On the other hand, an increasing trend of underweight was found in boys and girls (P < 0.0001). In adolescents aged to 16–17 years old, a stabilization was observed for the prevalence of overweight and obesity whereas the prevalence of underweight increased significantly in boys (Table 2). For adolescents aged to 10–12 and 13–15 years old, the prevalence of obesity and overweight decreased significantly while the proportion of underweight was increased significantly for boys and girls aged 10–12 years (Table 2). The prevalence of underweight increased in boys aged 13–15 years while that a stagnation was observed in girls in the same age range (Table 2).

**Table 2:** Overall and sex-specific number, proportions (n/%) children, age 10–17 years, classified as underweight, healthy weight, overweight and obese in 2008 and 2018 from the Diagnoform program(n = 90 250)

		2008		2009		2010	2011	2012	2013		2014		2015	2016		2017		2018		P for trend*
10–12 years																				
Underweight																				
Overall		646 (10.59)		218 (10.86)		495 (10.26)	542 (13.10)	349 (11.52)	278 (10.83)		395 (12.77)		208 (15.25)	384 (12.37)		741 (16.91)		351 (14.20)		** < 0.0001**
Boys		293 (9.29)		106 (9.80)		221 (8.64)	267 (11.22)	129 (8.41)	122 (9.36)		160 (10.30)		94 (13.54)	184 (11.16)		338 (15.06)		150 (11.99)		** < 0.0001**
Girls		353 (11.99)		112 (12.09)		274 (12.11)	275 (15.63)	220 (14.71)	156 (12.34)		235 (15.26)		114 (17.01)	200 (13.75)		403 (18.85)		201 (16.46)		** < 0.0001**
Healthy weight																				
Overall		4072 (66.77)		1442 (71.81)		3345 (69.37)	2861 (69.14)	2093 (69.10)	1789 (69.69)		2173 (70.23)		971 (71.19)	2156 (69.46)		3032 (69.18)		1738 (70.30)		**0.00921**
Boys		2161 (69.49)		779 (72.00)		1791 (69.99)	1684 (70.79)	1086 (70.84)	919 (70.53)		1127 (72.52)		501 (72.19)	1177 (71.38)		1559 (69.44)		908 (72.58)		**0.0485**
Girls		1911 (64.91)		663 (71.61)		1554 (68.67)	1177 (66.91)	1007 (67.31)	870 (68.83)		1046 (67.92)		470 (70.15)	979 (67.29)		1473 (68.90)		830 (67.98)		0.06781
Overweight																				
Overall		1044 (17.11)		271 (13.50)		756 (15.68)	566 (13.68)	471 (15.55)	381 (14.84)		432 (13.96)		157 (11.51)	464 (14.95)		512 (11.68)		302 (12.22)		** < 0.0001**
Boys		531 (16.83)		153 (14.14)		423 (16.53)	332 (13.95)	259 (16.89)	205 (15.74)		218 (14.03)		77 (11.10)	240 (14.55)		289 (12.87)		149 (11.91)		** < 0.0001**
Girls		513 (17.43)		118 (12.74)		333 (14.71)	234 (13.30)	212 (14.17)	176 (13.92)		214 (13.90)		80 (11.94)	224 (15.39)		223 (10.43)		153 (12.53)		** < 0.0001**
Obese																				
Overall		337 (5.53)		77 (3.83)		226 (4.69)	169 (4.08)	116 (3.83)	119 (4.64)		94 (3.04)		28 (2.05)	100 (3.22)		98 (2.23)		81 (3.28)		** < 0.0001**
Boys		170 (5.39)		44 (4.06)		124 (4.84)	96 (4.04)	59 (3.86)	57 (4.37)		49 (3.15)		22 (3.17)	48 (2.91)		59 (2.63)		44 (3.52)		** < 0.0001**
Girls		167 (5.67)		33 (3.56)		102 (4.51)	73 (4.14)	57 (3.81)	62 (4.91)		45 (2.62)		6 (0.90)	52 (3.57)		39 (1.82)		37 (3.03)		** < 0.0001**
13–15 years																				
Underweight																				
Overall		222 (10.05)		170 (9.80)		417 (10.56)	569 (13.05)	605 (11.41)	375 (10.26)		648 (11.98)		341 (13.94)	401 (11.68)		260 (13.81)		179 (11.00)		**0.0002**
Boys		103 (8.39)		91 (9.59)		188 (8.68)	295 (11.88)	232 (8.60)	186 (9.10)		253 (9.89)		132 (11.22)	180 (10.69)		121 (12.78)		79 (10.38)		**0.003**
Girls		119 (12.13)		79 (10.05)		229 (12.85)	274 (14.60)	373 (14.32)	189 (11.72)		395 (15.20)		209 (16.46)	221 (12.63)		139 (14.85)		100 (11.55)		0.115
Healthy weight																				
Overall	1681 (76.13)			1302 (75.04)		2992 (58.04)	3218 (73.79)	3883 (73.22)	2654 (72.59)		3868 (75.00)		1874 (76.61)	2601 (75.76)		1373 (72.91)		1278 (78.55)		0.255
Boys	928 (75.63)			702 (73.97)		1637 (75.54)	1823 (73.39)	1990 (73.76)	1467 (71.81)		1930 (75.42)		893 (75.93)	1264 (75.06)		679 (71.70)		590 (77.53)		0.255
Girls	753 (76.76)			600 (76.34)		1355 (76.05)	1395 (74.32)	1893 (72.67)	1187 (73.59)		1938 (74.60)		981 (77.25)	1337 (76.44)		694 (74.15)		688 (79.45)		0.217
Overweight																				
Overall	243 (11.01)			200 (11.53)		453 (11.47)	467 (10.71)	659 (12.43)	482 (13.18)		523 (10.13)		199 (8.14)	341 (9.93)		195 (10.36)		141 (8.67)		**0.011**
Boys	158 (12.88)			115 (12.12)		287 (13.24)	292 (11.75)	377 (13.97)	301 (14.73)		299 (11.68)		131 (11.15)	190 (11.28)		112 (11.82)		74 (9.72)		**0.011**
Girls	85 (8.66)			85 (10.81)		166 (9.31)	175 (9.32)	282 (10.82)	181 (11.22)		224 (8.62)		68 (5.35)	151 (8.63)		83 (8.87)		67 (7.73)		**0.003**
Obese																				
Overall	62 (2.81)			63 (3.63)		87 (2.20)	107 (2.45)	156 (2.94)	145 (3.97)		118 (2.29)		32 (1.31)	90 (2.62)		55 (2.92)		29 (1.78)		**0.037**
Boys	38 (3.10)			41 (4.32)		55 (2.54)	74 (2.98)	99 (3.67)	89 (4.36)		77 (3.01)		20 (1.70)	50 (2.97)		35 (3.70)		18 (2.37)		0.344
Girls	24 (2.45)			22 (2.80)		32 (1.80)	33 (1.76)	57 (2.19)	56 (3.47)		41 (1.58)		12 (0.95)	40 (2.29)		20 (2.13)		11 (1.27)		0.104
16–17 years																				
Underweight																				
Overall	253 (12.09)			62 (9.71)		189 (9.95)	168 (13.35)	154 (11.93)	158 (10.25)		292 (14.43)		233 (14.06)	308 (11.41)		127 (17.30)		184 (11.67)		**0.015**
Boys	108 (8.73)			28 (7.14)		81 (7.22)	68 (9.71)	53 (8.07)	79 (8.69)		125 (11.53)		93 (11.33)	138 (9.79)		84 (18.54)		92 (10.27)		** < 0.0001**
Girls	145 (16.96)			34 (13.76)		108 (13.88)	100 (17.92)	101 (15.93)	79 (12.50)		167 (17.78)		140 (16.75)	170 (13.17)		43 (15.30)		92 (13.52)		0.118
Healthy weight																				
Overall	1555 (74.33)			492 (76.99)		1481 (77.99)	924 (73.45)	954 (73.90)	1156 (75.02)		1501 (74.21)		1236 (74.59)	2002 (74.12)		499 (67.98)		1158 (73.43)		**0.003**
Boys	924 (74.70)			313 (79.85)		887 (79.13)	539 (77.00)	500 (76.10)	684 (75.25)		799 (73.70)		629 (76.61)	1040 (73.76)		296 (65.35)		659 (73.55)		** < 0.0001**
Girls	631 (73.80)			179 (72.47)		594 (76.35)	385 (69.00)	454 (71.61)	472 (74.68)		702 (74.76)		607 (72.61)	962 (74.51)		203 (72.24)		499 (73.27)		0.9568
Overweight																				
Overall	217 (10.37)			67 (10.48)		191 (10.06)	140 (11.13)	146 (11.30)	172 (11.16)		183 (9.05)		157 (9.47)	310 (11.48)		92 (12.52)		180 (11.41)		0.215
Boys	155 (12.53)			41 (10.46)		128 (11.42)	82 (11.71)	87 (13.24)	112 (12.32)		120 (11.07)		88 (10.72)	187 (13.26)		59 (13.02)		115 (12.83)		0.404
Girls	62 (7.25)			26 (10.53)		63 (8.10)	58 (10.39)	59 (9.31)	60 (9.50)		63 (6.71)		69 (8.25)	123 (9.53)		33 (11.74)		65 (9.54)		0.179
Obese																				
Overall	67 (3.21)			18 (2.82)		38 (2.00)	26 (2.07)	37 (2.87)	55 (3.57)		47 (2.32)		31 (1.87)	81 (2.99)		16 (2.18)		55 (3.49)		0.612
Boys	50 (4.04)			10 (2.55)		25 (2.23)	11 (1.57)	17 (2.59)	34 (3.74)		40 (3.69)		11 (1.34)	45 (3.19)		14 (3.09)		30 (3.35)		0.951
Girls	17 (1.99)			8 (3.24)		13 (1.67)	15 (2.69)	20 (3.15)	21 (3.32)		7 (0.75)		20 (2.39)	36 (2.79)		2 (0.71)		25 (3.67)		0.301
Total																				
Underweight																				
Overall	1121 (10.78)			450 (10.26)		1101 (10.32)	1279 (13.11)	1108 (11.52)	811 (10.45)		1335 (12.99)		782 (14.30)	1093 (11.83)		1128 (16.11)		714 (12.58)		** < 0.0001**
Boys	504 (8.97)			225 (9.28)		490 (8.38)	630 (11.32)	414 (8.47)	387 (9.10)		538 (10.35)		319 (11.85)	502 (10.58)		543 (14.89)		321 (11.04)		** < 0.0001**
Girls	617 (12.91)			225 (11.48)		611 (12.67)	649 (15.47)	694 (14.66)	424 (12.08)		797 (15.70)		463 (16.68)	591 (13.15)		585 (17.44)		393 (14.20)		** < 0.0001**
Healthy weight																				
Overall	7308 (70.28)			3236 (73.80)		7818 (73.27)	7003 (71.77)	6930 (72.01)	5599 (72.11)		7542 (73.41)		4081 (74.65)	6759 (73.16)		4904 (70.07)		4174 (73.54)		**0.018**
Boys	4013 (71.42)			1794 (74.04)		4315 (73.80)	4046 (72.74)	3576 (73.16)	3070 (72.15)		3856 (74.97)		2023 (75.18)	3481 (73.39)		2534 (69.53)		2157 (74.16)		0.686
Girls	3295 (68.93)			1442 (73.61)		3503 (72.63)	2957 (70.52)	3354 (70.83)	2529 (72.07)		3686 (72.60)		2058 (74.13)	3278 (72.93)		2370 (70.64)		2017 (72.86)		**0.001**
Overweight																				
Overall	1504 (14.46)			538 (12.27)		1400 (13.12)	1173 (12.02)	1276 (13.26)	1035 (13.33)		1138 (11.08)		513 (9.38)	1115 (12.07)		799 (11.41)		623 (10.98)		** < 0.0001**
Boys	844 (15.02)			309 (12.75)		838 (14.33)	706 (12.69)	723 (14.79)	618 (14.52)		637 (12.27)		296 (11.00)	617 (13.00)		460 (12.62)		338 (11.62)		** < 0.0001**
Girls	660 (13.81)			229 (11.69)		562 (11.65)	467 (11.13)	553 (11.68)	417 (11.88)		501 (9.87)		217 (7.82)	498 (11.08)		339 (10.10)		285 (10.30)		** < 0.0001**
Obese																				
Overall	466 (4.48)			158 (3.60)		351 (3.29)	302 (3.09)	309 (3.21)	319 (4.11)		259 (2.52)		91 (1.66)	271 (2.93)		169 (2.41)		165 (2.91)		** < 0.0001**
Boys	258 (4.59)			95 (3.92)		204 (3.49)	181 (3.25)	175 (3.58)	180 (4.23)		166 (3.19)		53 (1.97)	143 (3.01)		108 (2.96)		92 (3.16)		** < 0.0001**
Girls		208 (4.35)		63 (3.22)		147 (3.05)	121 (2.88)	134 (2.83)	139 (3.96)		93 (1.84)		38 (1.37)	128 (2.84)		61 (1.82)		73 (2.64)		** < 0.0001**

^*^Cochran–Armitage trend test. Significant P values are indicated in bold font

## Discussion

Updated information on the prevalence of the different weight categories is needed in order to properly develop, implement, and assess the effectiveness of the current public health initiatives and policies elaborated to fight unhealthy lifestyle in French adolescents. There is today a real need for actualized prevalence of underweight, overweight and obesity in French adolescents since the last available data were reported in 2013 [7, 8].

A pooled analysis on worldwide trends in BMI categories showed that the growing overweight and obesity prevalence trend had reached a plateau showing sometimes a slight decline in high-income countries [11]. Similar trends were observed among French adolescents with a stable prevalence of overweight only [7, 8]. A significant increase was however observed for adolescent obesity between 2009 and 2013 [8]. The results of the present study are very encouraging, suggesting for the first time a significant decline of the prevalence of overweight and obesity in French adolescents respectively decreasing from 14.5% to 11% and 4.5% to 2.9%. These changes are maybe due in part to the increased population awareness of this public health problem, as well as interventions promoting daily physical activity and healthy diets developed by national public health policies. Indeed, several studies showed in France, with a multi-level, long term community-based approach to childhood and adolescence obesity prevention, that the combined prevalence of overweight and obesity in youth decreased significantly [5, 12, 13]. However, the present results also show that the prevalence of overweight and obesity among adolescents aged between 16 and 17 years did not changed significantly. A plateau is observed at this age range. Therefore, current national public health initiatives in children and adolescents must be maintained and strengthened in order to continue to decrease the prevalence of obesity in youth, particularly in late adolescence.

Importantly, our finding also underline a gender effect. Indeed, the prevalence of overweight and obesity were higher in boys compared with girls (13.4% vs 11.1% for overweight boys and girls; 3.5% vs 2.8% for obese boys and girls). However, the prevalence remains high for both sexes. Our results concur with previous studies performed in France and others European or North American countries [8, 14, 15].

Some previous prevalence studies began to analyze the prevalence rate of underweight, highlighting another worldwide major health concern in children and adolescents [4, 8, 9]. Indeed, given health adverse consequences of underweight in youth, such as a poor quality of life, lower physical fitness, amenorrhea, decreased bone health, negative body image and fatigue, and, in later life, with increased mortality, it is needed to assess the rate underweight prevalence in future studies when monitoring overweight and obesity is performed [16]. In our study, results are particularly alarming with an increase of the proportion of underweight whatever the sex. As already pointed out by previous studies conducted in adolescents, a significant difference was found between boys and girls with a higher prevalence rate observed in girls (14.2% vs 10.2%) [4, 8, 9]. Another major outcome from our study was that the prevalence rate of underweight became superior to that of overweight in girls (14.2% for underweight vs 11.1% for overweight). These results underline the urgent need to develop strategies to reduce this growing prevalence, similarly to what is done for overweight and obesity.

In summary, our study shows a decline of the prevalence of overweight and obesity that remained however high between 2008 and 2018. The prevalence of underweight increased significantly. These results show the continued need to develop preventive and nutritional programs in order to modify the lifestyle for overweight, obese and underweight adolescent.

## Limitations

The large sample size with anthropometrics measurements (such as age and sex) was the main strength of our study. However, even though we collected data on a large sample, the study presents some limitations. The first limitation of this current study was to use a self-reported weight and height, a subjectively assessment methods. This method introduces systematic error because self-reports of weight and height are usually less and greater, respectively, than the corresponding measurements. Generally, values for weight are underreported, and low values for height are overreported [17]. Then, we cannot confirm that the cohort studied in our study is representative of French adolescents due to a bias selection. Indeed, the methodology of this program did not use a stratified sample design. In addition, due to the study design and voluntary programme, the number of adolescents across the years studied differs significantly and may affect our results. Lastly, the lack of information collected on socio-economic status, home location (urban, rural) and parental educational levels, and could have impacted our findings [6]. Indeed, previous studies showed that environmental or family factors, such as socio-economic status, were associated with the prevalence of overweight and obesity [7, 18]. Although it is more difficult in this type of study, future studies on the prevalence assessment could be perform using a more rigorous methodology such as including a random subject selection and to collect data about socio-economic status.

## Data Availability

The datasets used and/or analyzed during the current study are available from the corresponding author on reasonable request.

## Abbreviation

BMI: Body Mass Index
